# Healthcare Decision-Making in a Crisis: A Qualitative Systemic Review Protocol

**DOI:** 10.1155/2024/2038608

**Published:** 2024-04-12

**Authors:** Ehmaidy Al Qaf'an, Stewart Alford, Kimberley Porteous, David Lim

**Affiliations:** ^1^Centre for Improving Palliative, Aged and Chronic Care through Clinical Research and Translation (IMPACCT), Faculty of Health, University of Technology Sydney, Broadway, Ultimo, Australia; ^2^Kaplan Business School, Kaplan Australia, Brisbane, Australia; ^3^Education and Research Services (Health), UTS Library, University of Technology Sydney, Broadway, Ultimo, Australia; ^4^Mparntwe Centre for Evidence in Health: A JBI Centre of Excellence, Alice Springs, Australia

## Abstract

*Background*. Throughout history, communities have faced outbreaks of infectious diseases and other natural and man-made disasters that pose significant threats to lives, public health, and business continuity. Many of these disasters are crises that require critical decisions to be made in a short, crucial time with limited information and unforeseen circumstances amidst panic, fear, and shock. The COVID-19 pandemic is a recent example, with public leaders responding to and formulating strategies to attenuate the relentless waves of transmission and surges in resource demands. The pandemic underscored the importance of understanding how healthcare leaders make decisions in-crisis and what factors healthcare leaders prioritize in their decision-making process. *Methods/Design*. PubMed(NLM), Embase(Ovid), Scopus(Elsevier), Business Source(EBSCOhost), and ProQuest will be searched for primary qualitative studies published in English to explore the multi-faceted decision-making processes of healthcare leaders during a public health crisis. A meta-ethnographic approach will synthesize insights into healthcare leaders' experiences and perspectives and generate a conceptual theory of decision-making in crisis. *Discussion*. Understanding how healthcare leaders make critical decisions during public health crises takes advantage of the lessons learned to inform how future health crises are managed. (This systematic review is registered in PROSPERO: CRD42023475382).

## 1. Background

Health crises are complex, often unpredictable, and potentially destabilize society, as evidenced by the recent COVID-19 pandemic. Leadership quality can make a crucial difference between an effective response and chaos. While many studies have examined leadership styles in ordinary circumstances, leadership in health crises has received significantly less attention [1]. Some research indicates that certain leadership styles, such as transformational, transactional, ethical, and charismatic, can positively influence crisis management [2–5]. However, crises are complex, fast-paced, and involve high levels of uncertainty and pressure [6]. Critical decisions must be made quickly, and decision-makers must adjust and respond in various ways as a situation evolves and often dictates. Effective leadership in health crises can foster adherence to public health measures and win support for critical actions by creating trust [7]. Trust is essential since health crises affect individuals and communities emotionally and psychologically [8, 9]. Compassionate health leaders recognize the human impacts of the crisis and act to improve the mental health of impacted people [10]. The aftermath of a health crisis demands thoughtful planning and rebuilding [11, 12]. Understanding the importance of leadership in times of health crisis and encouraging the formation of such leadership quality will help navigate and resolve forthcoming health crises, assuring the well-being of the populations one serves.

As the Chinese characters for “crisis” (wéijī) indicate, a crisis has both positive and negative sides. While each crisis is unique, some common features of a “crisis” include major disruption to operations, negative public perception and strain on the individual, organization or system, and potential loss of morale and support [13]. Keown‐McMullan [14] proposed the anatomy of a “crisis” comprised of (i) the trigger for the crisis being perceived, (ii) the feeling of not being able to cope with the change that is taking place, and (iii) the trigger poses a threat to survival. The recent COVID-19 pandemic is an example of a community crisis affecting the whole of society when many of the previously known and successful public health containment strategies and disaster management processes for containing the 2003 SARS and other infectious disease epidemics were overwhelmed [15]. After the initial shock of the increased incidence of hospitalization and mortality in early 2020, the initial public health response included national lockdown, quarantine, and increased hospital bed capacity to cope with the surge in demands. The latter included cancelling elective surgery, transitioning to online and virtual healthcare, increasing the available workforce, including the deployment of ancillary staff and reserved workforce to frontline response, and reducing infection rates among the health workforce [16]. The public health decision had to be made regardless of the political priorities for economic and social freedom, uncertainty of information, and the emerging and evolving degree of population health risk. Other than the negatives from the pandemic response, some positives do emerge. These include the improved uptake of virtual healthcare [17], expanded and extended scope of health professional practice [18–21], appropriate planning and resourcing of essential services [22, 23], the importance of engaging with consumers, and appropriate culturally and linguistical public health approaches to underserviced communities [24–28]. Throughout the COVID-19 pandemic, rapid systematic reviews were conducted to synthesize evidence to inform the decision-making by public leaders. Recent published reviews on crisis leadership have focused on the event (natural crises [29] and organizational crises [30]), the leadership style (compassionate leadership [31]) and leadership elements [32, 33], the leadership competencies [34], and the roles that leaders play in a crisis [35] and post-crisis [36]. The bibliometric analysis of existing literature found a knowledge gap in the process view of crisis leadership and the contextualization of the crisis [37], as well as the importance of understanding how decisions are made [38].

This protocol outlines the methodology for conducting a qualitative systematic review of evidence related to the healthcare leaders' decision-making process during a crisis (“in-crisis”) with a specific focus on a public health crisis. Public health has a broader view of socioeconomic determinants and drivers than some of the conventional clinical medicine in general and has as its core principle the promotion and protection of health through coordinated partnership. Public healthcare leaders draw on tasks, people, and adaptive competencies influenced by political, structural, and cultural contexts [33]. All 194 member states of the World Health Organization (WHO) are currently negotiating an amendment to the existing international agreement and a new international future pandemic prevention, preparedness, and response instrument [39]. This review is timely and significant as it offers an organized method for compiling, examining, and combining existing evidence to generate a conceptual theory of how healthcare leaders make decisions in crisis and what factors leaders prioritize in their decision-making process. Utilizing a structured and rigorous systematic review process, lessons could be learned from past experiences to improve knowledge of leadership functions in health crises and be better prepared for future public health crises. Knowing what intrinsic and extrinsic factors influenced healthcare leaders' decision-making process in a public health crisis would better inform professional development or training for the current and emerging healthcare leaders to be better prepared for making tough decisions in a short, crucial time with limited information amidst panic, fear, and shock. System changes may be made—such as what data to collect, how data would be interpreted or used, and how advancements in technology, such as artificial intelligence, could be harnessed—to better assist with decision-making as a public health crisis evolves. Organizations and leaders can enhance their crisis response plans by adopting and modifying best practices. Previous health crises have yielded valuable lessons for leadership; this systematic review allows for the compilation and analysis of these lessons learned from various sources and settings to avoid repeating mistakes and capitalize on successful approaches.

## 2. Methods/Design

### 2.1. Review Question

How do healthcare leaders make decisions in-crisis, and what factors do they prioritize in their decision-making process?

### 2.2. Systemic Review

The review is registered with PROSPERO (CRD 42023475382).

## 3. Eligibility Criteria

### 3.1. Inclusion and Exclusion Criteria

- Qualitative studies include but are not limited to study designs such as phenomenological, ethnographic, grounded theory, historical and case studies. Primary studies included in secondary studies (e.g., systematic review, rapid review, and narrative review) will be considered. Backward citation searching will be conducted manually by inspecting the reference list of the source study per the JBI methods for qualitative systematic review [40]. Grey literature will also be considered for inclusion. These unconventional resources include reports, board meetings, and conference proceedings. The research team has experience tapping into organizational databases, government archives, and specialized online platforms in other policy-related reviews conducted [41, 42].
- Published in English.
- To be comprehensive, this review will consider all published work since 1962 to be concordance with and extend the recently published bibliometric analysis (1962–2020) on leadership and decision-making under crisis [38].

Our search terms, informed by the recently published qualitative systematic review on crisis management [34], are presented in Table 1.

### 3.2. Type of Participants

All health leaders, managers, and administrators with leadership responsibilities and positions involving organizational or system decision-making are included in the study.

### 3.3. The Information Sources

The databases to be searched include PubMed(NLM), Medline(Ovid), Embase(Ovid), Scopus(Elsevier), Business Source(EBSCOhost), CINAHL(EBSCOhost), and ProQuest. The reference list of related secondary studies and all papers included will be screened for additional studies. Peak organizations' homepages, such as the World Health Organization, Centers for Disease Control and Prevention, International Federation for Emergency Medicine, World Association for Disaster and Emergency Medicine, and United Nations, would also be hand-searched for relevant papers.

### 3.4. Study Selection

On completion of the search, hits from each database will be imported into the cloud-based Covidence software for record management. After removing duplicates, titles and abstracts will be screened against the inclusion criteria independently by two reviewers (EA and SA). Full-text papers that do not meet the inclusion criteria will be excluded, and the final report will provide reasons for exclusion. The final report will report the search results and present a Preferred Reporting Items for Systematic Reviews and Meta-Analyses (PRISMA) flow diagram [43]. Any disagreements between the reviewers will be resolved through discussion or with a third reviewer (SA or DL).

### 3.5. Assessment of Methodological Quality

Two reviewers (EA and SA) will independently evaluate the methodological validity of the included papers using the JBI critical appraisal instrument. Any discrepancies between the reviewers will be settled through discussion or with a third reviewer (DL). Findings of the critical appraisal will be reported narratively and tabulated. Kappa statistics will be calculated for levels of agreement.

### 3.6. Data Extraction

Data extraction will be conducted in Covidence (EA or SA). The extracted data will include participants, study methods, context, types of health crises, what factors did healthcare leaders considered in the decision making in-crisis, and process or theory of decision making in-crisis. DL would perform a random audit of the data extraction.

### 3.7. Data Synthesis

Qualitative findings will be pooled using Noblit & Hare 7-step meta-ethnography [44] to capture the collective shared understanding and experience of healthcare leaders making decisions in crisis. The rationale for employing this method lies in its ability to go beyond a simple aggregation of findings and provide a more nuanced and comprehensive understanding of a particular phenomenon. The steps are as follows: (i) identification of an issue needing further investigation, (ii) deciding what is relevant, (iii) careful reading and coding of the selected studies, (iv) determining how the studies are related through the grouping of codes into themes iteratively, (v) translating the studies into one another using reciprocal translational analysis, refutational synthesis and the generation of lines-of-argument synthesis, (vi) synthesizing the translations by searching for overarching explanations and identifying gaps and overlaps, and (vii) expressing the synthesis using the eMERGe guideline [45] and ConQual checklist to establish confidence in the synthesis. Extraction and translation of studies will be conducted in NVivo software, which enables the reviewers to access the original papers easily and provides an auditable trail. The conceptual theory of how healthcare leaders make decisions in crisis will be validated by three informed insiders whom have experiences with public health crises. Feedback from the informed insiders would be used to inform and revise the syntheses [46].

## 4. Discussion

Effective decision-making by healthcare leaders during a public health crisis is critical since it mitigates the impact of public health crises. Effective decision-making is essential for protecting public health, optimizing resource utilization, and ensuring communities receive the necessary care and support during challenging times. The decisions made by healthcare leaders during a crisis can affect a wide range of crisis-related issues, including but not limited to resource allocation, workforce management, treatment protocols, communication strategies, public health initiatives, and community outreach and support. This systemic review explores how healthcare leaders prioritize critical decisions amid a crisis.

## 5. Conclusion

Although public health crises impose a drastic burden on society and the individual, effective decision-making by healthcare leaders can act to minimize harm, saving the lives and livelihoods of entire communities. While the current literature explores crisis leadership styles in general circumstances, this qualitative systematic review endeavours to synthesize the available evidence concerning the public health context. In this manner, this review will fill a critical knowledge gap while empowering the healthcare leaders of tomorrow to make well-informed decisions through structured processes with clear priorities, allowing for improved health crisis management in the face of ongoing and future public health challenges. This systematic review will interest researchers, health leaders, senior managers, team leaders, and education enterprises.

## Figures and Tables

**Table 1:** Search terms.

PICo	Inclusion	Exclusion	Search terms
Participants (P)	Healthcare leaders	Health professionals not in a leadership role or involved in organizational or system decision-making positions; health students	“Healthcare leader^*∗*^”[tiab] OR “healthcare executive^*∗*^”[tiab] OR “healthcare administrator^*∗*^”[tiab] OR “medical leader^*∗*^”[tiab] OR “clinical leader^*∗*^”[tiab] OR “hospital administrator^*∗*^”[tiab] OR “health system leader^*∗*^”[tiab] OR “healthcare manager^*∗*^”[tiab]

Phenomenon of interest (I1)	Experiences or reflection on healthcare leadership	Leadership outside of healthcare	“Leadership strategy^*∗*^”[tiab] OR “strategies in leadership”[tiab] OR “leadership approach^*∗*^”[tiab] OR “effective leadership”[tiab] OR “successful leadership”[tiab] OR “leadership style^*∗*^”[tiab] OR “strategic leadership”[tiab] OR “leadership tactic^*∗*^”[tiab] OR “innovative leadership”[tiab] OR “adaptive leadership”[tiab] OR “transformational leadership”[tiab] OR “situational leadership”[tiab] OR leadership[Mh] OR “experience^*∗*^”[tiab] OR “reflect^*∗*^”[tiab]

Phenomenon of interest (I2)	Decision-making	Decision-making outside of healthcare	“Emergency decision-making”[tiab] OR “disaster decision-making”[tiab] OR “healthcare crisis decision^*∗*^”[tiab] OR “medical emergency decision^*∗*^”[tiab] OR “hospital crisis management”[tiab] OR “decision strategies in crises”[tiab] OR “decision processes during emergency^*∗*^”[tiab] OR “decision-making in critical situations^*∗*^”[tiab] OR “decision-making process”[tiab] OR “decision processes”[tiab] OR “decision-making mechanism^*∗*^”[tiab] OR “decision modelling”[tiab] OR “decision-making pathway^*∗*^”[tiab] OR “medical decision-making”[tiab] OR “healthcare decision-making”[tiab] OR “clinical decision processes”[tiab] OR decision-making[Mh] OR mental processes[Mh]

Context (Co1)	Healthcare, public health, or population health	Nonhealthcare organizations, universities and other educational institutions	“Hospital”[tiab] OR “community”[tiab] OR “medical care”[tiab] OR “medical industry”[tiab] OR “public health”[tiab] OR “population health”[tiab] OR health[Mh] OR organizations[Mh] OR delivery of health care[Mh] OR tertiary healthcare[Mh] OR primary health care[Mh] OR patient care management[Mh]

Context (Co2)	In-crisis or during a crisis	Precrisis or postcrisis	“Emergency response”[tiab] OR “emergency preparedness”[tiab] OR “emergency management”[tiab] OR “disaster response”[tiab] OR “disaster preparedness”[tiab] OR “disaster management”[tiab] OR “healthcare emergency response”[tiab] OR “medical emergency preparedness”[tiab] OR “hospital emergency management”[tiab] OR “crisis response”[tiab] OR “crisis preparedness”[tiab] OR “crisis management”[tiab] OR “crisis management”[tiab] OR “emergency management” OR “disaster management”[tiab] OR “Crisis”[tiab] OR critical care[Mh]

## Data Availability

The data used to support the study's findings of this study are available upon reasonable request to corresponding authors.

## Abbreviations

WHO: World Health Organization
JBI: Joanna briggs institute
PRISMA: Preferred reporting items for systematic reviews and meta-analyses
MESH: Medical subject headings
PROSPERO: The international prospective register of systematic reviews.
